# Psychological and Behavioral Factors Involved in Temporomandibular Myalgia and Migraine: Common but Differentiated Profiles

**DOI:** 10.3390/ijerph20021545

**Published:** 2023-01-14

**Authors:** Ana Cristina Viñals Narváez, Teresa Sánchez-Sánchez, Maria García-González, Ignacio Ardizone García, Rosana Cid-Verdejo, Eleuterio A. Sánchez Romero, Laura Jiménez-Ortega

**Affiliations:** 1 Department of Psychobiology and Behavioral Sciences Methods, Faculty of Odontology, Complutense University of Madrid, 28040 Madrid, Spain; 2 Department of Conservative and Prosthetic Dentistry, Faculty of Odontology, Complutense University of Madrid, 28040 Madrid, Spain; 3 Department of Clinical Dentistry, Faculty of Biomedical Sciences, Universidad Europea de Madrid, 28670 Madrid, Spain; 4 Department of Physiotherapy, Faculty of Sport Sciences, Universidad Europea de Madrid, 28670 Villaviciosa de Odón, Spain; 5 Musculoskeletal Pain and Motor Control Research Group, Faculty of Sport Sciences, Universidad Europea de Madrid, 28670 Madrid, Spain; 6 Department of Physiotherapy, Faculty of Health Sciences, Universidad Europea de Canarias, 38300 Santa Cruz de Tenerife, Spain; 7 Musculoskeletal Pain and Motor Control Research Group, Faculty of Health Sciences, Universidad Europea de Canarias, 38300 Santa Cruz de Tenerife, Spain; 8 Physiotherapy and Orofacial Pain Working Group, Sociedad Española de Disfunción Craneomandibular y Dolor Orofacial (SEDCYDO), 28009 Madrid, Spain; 9 Center of Human Evolution and Behavior, UCM-ISCIII, 28029 Madrid, Spain; 10 Psychology and Orofacial Pain Working Group, Sociedad Española de Disfunción Craneomandibular y Dolor Orofacial (SEDCYDO), 28009 Madrid, Spain

**Keywords:** temporomandibular disorders, migraine, psychological factors, stress coping, anxiety, depression

## Abstract

Background: Many studies have revealed high comorbidity and a clear association between temporomandibular disorders (TMD) and migraine. Furthermore, evidence points out that common psychological and behavioral factors might be related to the observed TMD and migraine association. However, this association and the underlying psychological factors are poorly understood. Objective: The main goal of this study was to describe the psychological and behavioral factors involved in TMD myalgia and migraine. Methods: A sample of 142 participants were recruited to form 4 groups: migraine patients (ICHD-III criteria), painful-TMD patients (Myalgia DC/TMD criteria), patients suffering from both pathologies according to the same criteria, and control patients. After a dental and neurological examination, the patients filled several psychological questionnaires validated for the Spanish population to assess anxiety (STAI), depression (DEP), stress coping (CRI), and somatic, anxiety, and depression symptoms (BSI-18). Results: The TMD myalgia patients, in general, showed a state of elevated anxiety, somatization, and reduced coping strategies, while the patients with migraine presented greater anxiety symptoms, depression (dysthymia trait and state), and somatization. Conclusions: According to the data of the present study, situational anxiety (transient emotional state), together with the lack of coping strategies, could be more associated with TMD myalgia, while anxiety, as a more stable and long-lasting emotional state, together with depression, might be more related to migraine. Further longitudinal studies are needed to unravel whether these differentiated profiles are a consequence or possible risk factors for migraine and TMD.

## 1. Introduction

Temporomandibular disorders (TMD) and migraine are significant public health problems and often appear associated. However, this association is still poorly understood and may be due to multiple biopsychosocial factors such as shared physiology, genetics, psychological traits, and environmental influences [1].

On the one hand, TMD include several clinical problems that involve the masticatory musculature, the temporomandibular joint, and associated structures [2]. The prevalence of TMD varies according to the sources consulted, possibly due, among other reasons, to the diagnostic criteria followed in each study [3,4,5]. According to the American Academy of Orofacial Pain [6], the prevalence of painful masticatory muscle disorders is 13%, that of disc disorders is 16%, and that of painful TMJ disorders is 9%. Due to the complexity of the masticatory system, TMD symptoms may be caused by different physiological and/or psychosocial factors [7,8]. On the other hand, migraine is a chronic neurological disorder characterized by recurrent episodes of headache and associated symptoms (e.g., nausea, sensitivity to light and noise) that typically last from 4 to 72 h. The migraine episodic manifestations are progressive in some individuals [9]. While headache has a prevalence of about 50% in the general population [10], migraine associated with TMD has a prevalence of about 14% in the population according to De Leeuw et al. [6] and Di Paolo et al. [10].

TMD and headaches are closely related pathologies, with a prevalence of headaches in the TMD population of about 67% [11]. Primary headaches such as migraine are more common in patients with TMD symptoms [12]. Additionally, patients with headaches and TMD (both arthralgia and myalgia) have significantly higher levels of pain and disability in comparison to patients with only TMD [9]. Although many studies demonstrate an association between TMD and headache, the underlying reasons for this association are poorly understood [13,14,15]. In recent years, some studies have shown a more specific relation between TMD and primary headaches and consider them as different entities that act on each other as perpetuating, aggravating factors [16], increasing the probability of progression to a chronic condition [17]. This topic was also addressed by the well-known OPPERA study, where the authors confirmed that migraine, but not tension-type headache, is a risk factor for incident TMD and that the association between migraine and TMD may be due to multiple biopsychosocial factors, such as shared physiology, genetics, psychological traits, and environmental influences [11,18]. 

Additionally, the OPPERA study also developed a heuristic model of the etiology of TMD, where associations between TMD and the psychosocial measures represented in the intermediate phenotype of “psychological suffering” were presented [18]. Other authors explained that global psychological and somatic symptoms emerged as the most robust risk factor for the incidence of TMD [19,20]. Su et al. [20] observed that the best predictor of pain intensity in TMD was somatization, while depression was the best predictor of pain-related disability. These findings indicate that there are multiple underlying psychological constructs and a substantial need to document the relationship between pre-existing psychological characteristics and the onset and persistence of TMD [5]. 

Although several risk factors are involved in migraine, such as gender, age, genetics, educational level, obesity, caffeine intake, etc. [21], evidence also indicates an association between chronic migraine and psychological factors [22,23]. Psychological and behavioral interventions in combination with pharmacotherapy are useful in migraine treatment [24]. Furthermore, comparably to TMD, migraine patients present higher levels of anxiety, depression, and neuroticism compared to controls [25,26]. In this line, it was found that TMD patients with anxiety or depression had a higher chance of developing migraine, using the Hospital Anxiety and Depression Scale (HADS) [13]. Additionally, it was observed that patients with TMD had a significantly higher prevalence of depression, which was more marked in patients with combined migraine and tension-type headache [27]. 

Overall, we can raise the concern that psychological factors, as an intrinsic part of an individual, are comorbid with these chronic pathologies and might influence their condition [28]. Therefore, it seems necessary to study the triad psychological factors–TMD myalgia–migraine and the relationships between the three, emphasizing the differentiation of the groups studied. Therefore, the main goal of this study was to describe the psychological and behavioral factors involved in TMD myalgia and migraine. To this aim, the psychological and behavioral factors influencing Spanish patients suffering from migraines, patients with painful muscle TMD (myalgia), patients with both pathologies, as well as control patients were examined. According to the above premises, one would expect to observe common psychological factors involved in both pathologies, such as anxiety and depression and maladaptive coping, but also specific psychological profiles for each pathology. 

## 2. Materials and Methods

### 2.1. Study Design

An observational, cross-sectional study was conducted between January 2018 and July 2018, recruiting 103 patients using a non-probabilistic method of judgmental or purposive sampling at two sites: the TMD and Orofacial Pain Clinic of the Complutense University of Madrid and the Headache Unit of the Modesto Lafuente Specialty Center (Hospital Clínico San Carlos). In accordance with the Declaration of Helsinki, all patients signed an informed consent form prior to inclusion and agreed to have their clinical information published anonymously. The study was approved by the Ethics Committee of the Hospital Clínico San Carlos, Madrid, Spain (Reference: 15/159-E).

### 2.2. Sample and Physical Examination

A sample of 142 participants from Spain (28 males, 103 patients, 38 asymptomatic subjects) between 18 and 65 years of age was consecutively recruited to form 4 groups. The TMD group (TMD+/Migraine−, *n* = 23) was selected among the Orofacial Pain clinic patients, which were diagnosed with painful muscle TMD (myalgia) according to DC/TMD criteria [3,29] but did not suffer migraines in the 6 months before the study. The migraine group (TMD−/Migraine+, *n* = 20) was composed of patients with a migraine diagnosis that did not present any associated secondary headache, following the ICHD-III guidelines [30]. Patients diagnosed with both migraine and TMD myalgia were included in a third group (TMD+/Migraine+, *n* = 60). The control group (TMD−/Migraine−, *n* = 39) was formed by patients’ companions that did not suffer any type of headaches (even mild ones) or TMD disorders in the 6 months before the interview. All patients were clinically assessed by a neurologist and a dentist who specialized in migraine and TMD, according to the ICHD-III and DC/TMD guidelines, respectively, and by the authors of this study. The participants were recruited during their first appointment; therefore, during questionnaire filling and the clinical explorations they did not receive any treatment either for migraine or for TMD. 

Only patients with painful muscle disorders (i.e., diagnosed with myalgia) were included in the study, firstly for the reliability of DC/TMD for the diagnosis of muscle disorders, and secondly, because it was not possible to confirm joint disorders with imaging diagnosis. 

The exclusion criteria for all groups were: pregnancy, alcohol abuse or drug use, patients undergoing medical treatment with opiate medications, history of severe psychiatric illnesses, history of surgery or trauma in the cranial or cervical region, concomitant severe systemic diseases, any active dental treatment at the time of the study, including orthodontic treatment, or inability to fill out the questionnaires.

### 2.3. Sample Size

The sample size was initially calculated with the method described by Naing et al. [31], assuming a prevalence of 7% and 14% (see the introduction for ranges) for migraine and DTM, respectively, with a level of confidence of 0.95 and a precision of 0.1. Sample size calculation resulted in 25 and 37 participants for migraine and DTM. Additionally, considering that the study was multivariate, to better estimate sample size adequacy for each variable, the power (θ) for each one was calculated.

### 2.4. Evaluation of Psychological, Cognitive, and Emotional Factors 

After the neurological and dental examinations, the patients filled a battery of psychological questionnaires. All selected tests are validated for a Spanish sample (Spain), are commonly used in research, and have high levels of reliability (Cronbach alphas between 0.59 and 0.93) and validity (correlations generally above 0.70) [32]. 

#### 2.4.1. Anxiety Assessment

Anxiety was measured using the STAI [32]. It is composed of 10 items assessing state anxiety STAI-State (transient emotional state), and another 10 items for trait anxiety STAI-Trait (anxious, relatively stable propensity of the participant in general). 

#### 2.4.2. Depression Assessment

The ST-DEP was used to assess depression [33]. This questionnaire includes depression scales for state and trait depression, and each one includes a euthymia (such as anhedonia) and a dysthymia subscale (negative mood, thoughts, and feelings).

#### 2.4.3. Psychopathological Symptoms Assessment

To further assess the symptoms of depression, anxiety, and somatization, the BSI-18 questionnaire [34] was also included. It is a brief self-applied inventory of psychopathological symptoms consisting of 18 items belonging to the SCL-90 family [35]. 

#### 2.4.4. Stress Coping Assessment

Stress coping was measured using the Coping Responses Inventory—Adult Form (CRI) [36]—which contains 48 items grouped into 8 scales: logical analysis (LA), positive reappraisal (PR), seeking guidance and support (SG), problem solving (PS), cognitive avoidance (CA), acceptance or resignation (A/R), seeking alternative rewards (SAR), and emotional discharge (ED). These 8 scales are classified according to two dimensions identified in the literature: Cognitive and Behavioral coping on the one hand and Approach vs. Avoidance coping on the other hand [37]. 

### 2.5. Statistical Analysis

After data collection, the results obtained were analyzed using the IBM SPSS 27 and R. To test normality the Henze–Zirkler’s Multivariate test was used [38] for the questionnaire scores of TMD+/Migraine−, TMD−/Migraine+, TMD+/Migraine+, and control groups (HZ = 0.999, *p* = 0.1; HZ = 0.999, *p* = 0.33; HZ = 0.999, *p* = 0.2, HZ = 0.999, *p* = 0.11, respectively). The results indicated that normality could be assumed to perform a 2 × 2 MANOVA analysis including the factors TMD (+/−) and Migraine (+/−) to compare the four groups of participants including all psychological variables. 

## 3. Results

After excluding 17 participants because of incomplete questionnaires or lack of relevant information, 142 participants were included in the study (28 males, 103 patients, 38 asymptomatic subjects). They were between 19 and 65 years old (mean age = 38.34; SD = 11.21). 

After the clinical examinations, the participants were classified into four groups: Migraine−/TMD− (*n* = 39), TMD+/Migraine− (*n* = 23); TMD−/Migraine+ (*n* = 20), and TMD+/Migraine+ (*n* = 60). Overall, the number of migraine participants, regardless of TMD, was 80, while the TMD myalgia participants, regardless of migraine, were 83. The characteristics of each sample group are described in Table 1.

Although the number of males and females between the groups was different, a Kruskal–Wallis gender proportion analysis did not produce significant differences between the groups (χ^2^ = 4.6, *p* = 0.21). In contrast, significant differences were observed for age (F(1,138) = 3.6; *p* < 0.05). Post-hoc analyses found a significance when comparing the mean age of the TMD−/Migraine+ and TMD+/Migraine+ groups (Δ = −7.9; *p* < 0.05). Finally, when comparing the chronic migraine prevalence between TMD−/migraine+ and TMD+/Migraine+ patients, significances also appeared (χ^2^ =13, *p* < 0.01).

Only the significant results are briefly described below; the detailed results and those from the post-hoc analyses can be found in Supplementary Material Tables S1 and S2, respectively.

### 3.1. Anxiety (STAI Questionnaire)

**Figure 1:**
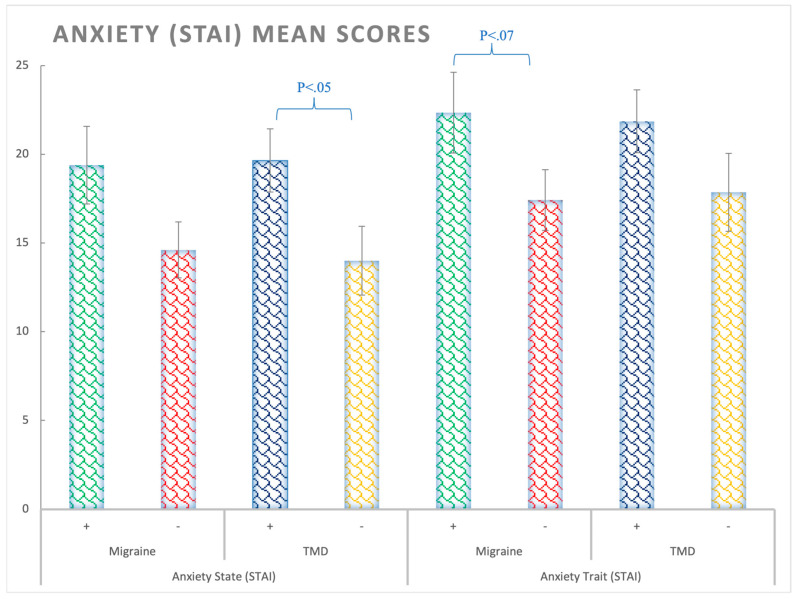
Anxiety (STAI) mean scores for state and trait subscales considering the Migraine (+,−) and TMD (+,−) Factors. Bars represent standard errors. STAI-Trait: No significant differences were observed for trait anxiety (Fs < 3.24, *p*s > 0.05); however, a trend to significance was observed for the migraine patients (F(1,138) = 3.24; *p* = 0.06; η_p_^2^ = 0.02; θ = 0.43).

STAI-State: Significant differences were observed in state anxiety for the TMD factor (F(1,138) = 4.9; *p* < 0.05; η_p_^2^ = 0.035; θ = 0.6), with patients with TMD presenting higher levels of anxiety than participants without TMD, regardless of migraine (Figure 1 and Supplementary Table S1).

### 3.2. Depression (ST/DEP Questionnaire) 

**Figure 2:**
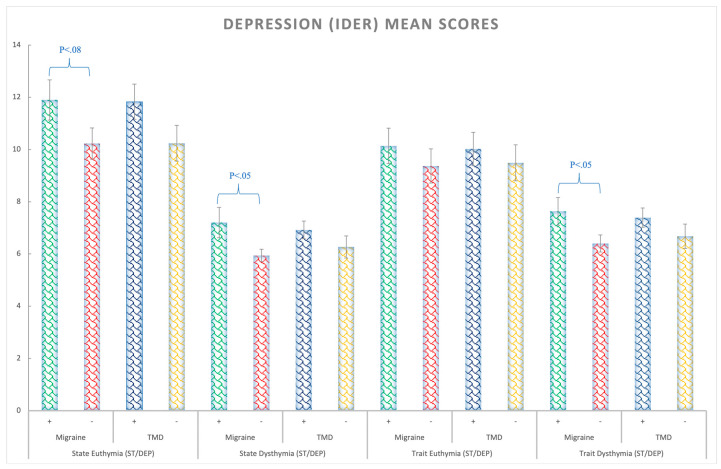
Depression (IDER) mean scores for the euthymia (similar to anhedonia) and dysthymia (negative mood, thoughts, and feelings) state and trait subscales considering the Migraine (+,−) and TMD (+,−) Factors. Bars represent standard errors.

Depression State: the migraine patients presented higher levels of state depression (F(1,138) = 4.52; *p* = 0.03; η_p_^2^ = 0.032; θ = 0.56) than the rest of the groups regardless of TMD, that is, for the migraine factor (Figure 2).

Depression Trait: A trend was found for trait depression *p* = 0.063 (all Fs < 3.51; *p*s > 0.05). Nonetheless, for the dysthymia subscale, the migraine patients showed a higher level of depression than the non-migraine participants (F(1,138) = 5.26; *p* = 0.023; η_p_^2^ = 0.04; θ = 0.62), regardless of TMD (Figure 2 and Supplementary Table S1).

### 3.3. Somatization, Depression, and Anxiety Symptoms (BSI-18)

**Figure 3:**
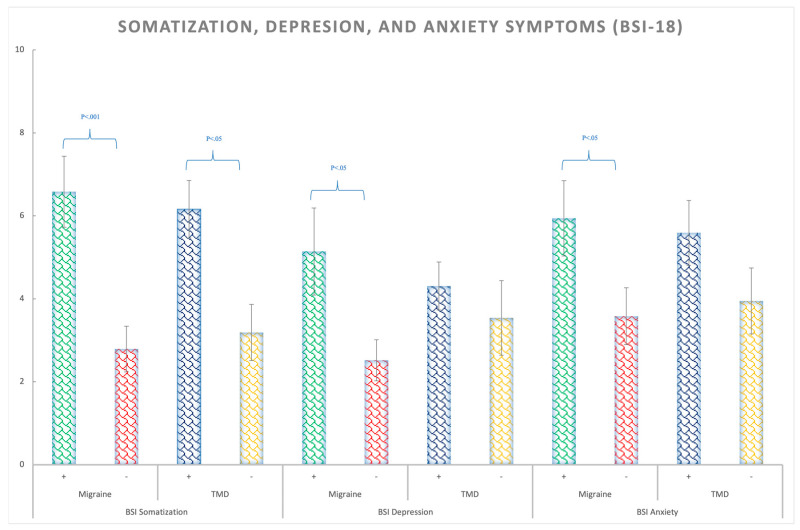
BSI-18 means scores for the somatization, depression, and anxiety symptoms in Migraine (+,−) and TMD (+,−) patients. Bars represent standard errors.

The migraine patients presented more somatization symptoms (F(1,138) = 14.43; *p* < 0.01; η_p_^2^ = 0.095; θ = 0.97), depression symptoms (F(1,138) = 8.59; *p* = 0.004; η_p_^2^ = 0.06; θ = 0.83), anxiety symptoms (F (1,138) = 4.74; *p* = 0.031; η_p_^2^ = 0.033; θ = 0.58), as well as a higher GSI (global severity index) (F(1,138) = 10.49; *p* = 0.002; η_p_^2^ = 0.07; θ = 0.89) than the non-migraines participants, regardless of TMD presence (Figure 2 and Supplementary Table S1). The BSI-18 test also indicated that the TMD patients, regardless of migraine, showed exclusively significantly larger scores for somatization (F(1,138) = 4.78; *p* = 0.03; η_p_^2^ = 0.033; θ = 0.58) (Figure 3 and Supplementary Table S1).

### 3.4. Stress Coping Styles (CRI)

**Figure 4:**
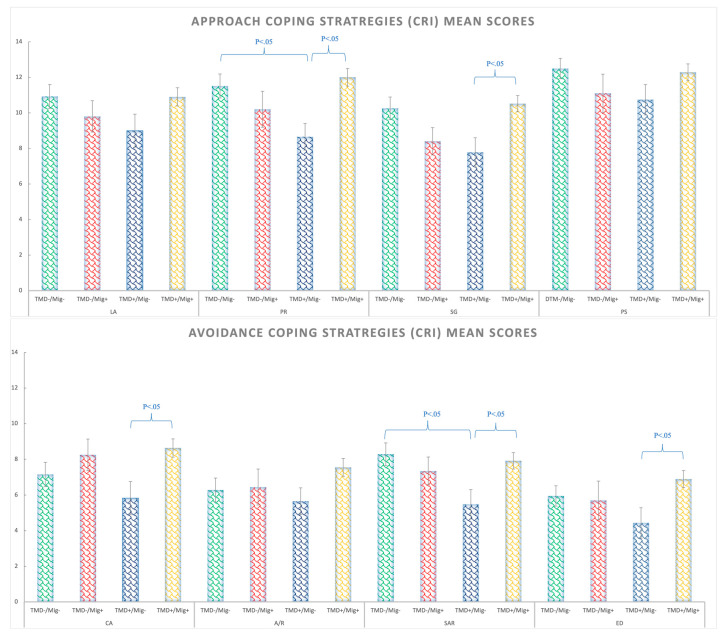
Coping strategies (CRI) mean scores for approach strategies (above) and avoidance strategies (bellow) in the TMD−/Migraine−, TMD−/Migraine+, TMD+/Migraine−, TMD+/Migraine+ groups. Abbreviations: logical analysis (LA), positive reappraisal (PR), seeking guidance and support (SG), problem solving (PS), cognitive avoidance (CA), acceptance or resignation (A/R), seeking alternative rewards (SAR), and emotional discharge (ED).

With respect to the stress coping styles, five out of the eight strategies showed significant migraine by TMD interactions (see Supplementary Table S1 and Figure 4 for details). For positive reappraisal (PR), the TMD+/Migraine− group presented fewer PR strategies than the control group (Δ = −2.86; *p* = 0.037) and the TMD+/Migraine+ group (Δ = −3.34; *p* < 0.05). Regarding the seeking guidance strategy (SG), the patients with just TMD (TMD+/Migraine−) used this strategy less than the TMD+/Migraine+ group (both pathologies) (Δ = −2.73; *p* < 0.05) and the control group (Δ = −2.5; *p* < 0.05). For cognitive avoidance (CA), the patients with just TMD (TMD+/Migraine−) presented lower levels than the TMD+/Migraine+ group (Δ = −2.8; *p* < 0.05). For seeking alternative reward (SAR), the results showed that the group with TMD without migraine (TMD+/Migraine−) presented significantly less SAR than the control group (Δ = −2.8; *p* < 0.05) and the TMD+/Migraine+ group (Δ = −2.43; *p* < 0.05). Finally, emotional discharge (ED) was lower in the TMD+/Migraine− compared to the group with both TMD and the migraine group (TMD+/Migraine+) (Δ = −2.46; *p* < 0.05). Detailed post-hoc analyses can be found in Supplementary Material (Table S2).

## 4. Discussion

The reported data indicate that TMD myalgia patients, in general, showed an elevated state of anxiety and somatization, and reduced coping strategies, while patients with migraine presented more intense anxiety symptoms, depression (dysthymia trait and state), and somatization.

As detailed above, higher levels of state anxiety observed in temporomandibular myalgia patients were observed, compared to patients without TMD, regardless of migraine. However, these patients presented neither elevated trait anxiety (STAI-trait) nor anxiety symptoms (BSI-18) in comparison to controls. Previous studies, using the STAI questionnaire, showed contradictory results. An association between both state and trait anxiety with TMD was observed in participants with a low quality of life and several comorbidities [39], while in a sample of students, significant differences were not found for trait anxiety, similar to our results [40]. The severity and type of painful TMD (myalgia vs. arthralgia), the sample characteristics, and the migraine comorbidity might explain such contradictory findings. According to our data, when the presence of migraine is controlled, there exists a relation between state anxiety (more situational anxiety) and TMD, myalgia but not a relation between TMD myalgia and trait anxiety, which is a more stable characteristic of personalities prone to suffer anxiety [32]. Likewise, in the present study, no significant differences were found in anxiety symptoms (BSI-18) in TMD myalgia patients in comparison to controls. In this case, previous experiments using similar measurements, such as SCL-90, also showed varied results [7,20,41,42,43]. This variety of results might be similarly explained by the TMD severity and type, the sample characteristics, and/or possible comorbidities.

The migraine patients presented significantly greater anxiety symptoms (BSI-18) and nearly statistically significant levels of anxiety (STAI-trait) compared to participants without migraine, regardless of TMD, although no significant differences were found in state anxiety. The role of anxiety in migraine is documented and is in line with our results. For example, Zampieri et al. [44] observed that patients with chronic migraine presented high levels of anxiety. In a recent study using also the STAI questionnaire, it was also found that the chronic migraine group showed greater trait anxiety and general anxiety sensitivity compared to the control group [22]; however, these authors did not control for migraine presence. Other authors such as Nazeri et al. [13] studied the role of anxiety in patients with migraine and TMD through the HADS questionnaire. In line with our results, they found that TMD patients with anxiety or depression were more likely to suffer from migraine. Summarizing the anxiety findings, our data indicate that anxiety is present in both TMD and migraine patients; however, situational anxiety is observed in TMD patients, while a more stable trait of anxiety accompanied by anxiety symptoms is found in migraine patients.

Moving to the depression variables for both scales (state and trait dysthymia depression) of the IDER questionnaire and BSI-18 symptom depression scale, the migraine patients showed more depression symptoms, unlike patients with TMD and controls. In this line, Ballegaard et al. [27] observed that people with moderate to severe depressive symptoms were also more likely to develop a headache. Antonaci et al. [25] argued that depression is almost twice as frequent in subjects with migraine, and several authors claim that depression might be a risk factor for migraine, also pointing out that the association between migraine and depression appears to be bidirectional [13,26,45,46,47]. 

Concerning TMD myalgia and depression, unlike some previous references [7,42,43], our patients with TMD did not show higher levels of depression in the IDER or the BSI-18 compared to the control group when controlling for migraine. This could be in line with the OPPERA study [18] that established the “presence of a negative state of mind” as a risk factor for TMD or a predictor, but not depression itself. A negative state of mind, although related to depression, might be milder and does not exactly correspond to it.

Regarding somatization, in our study, significant differences were observed for both migraine and TMD myalgia patients, regardless of comorbidities. Most of the somatization studies used the SCL 90-R test [48] to measure somatization; since the BSI-18 is a brief validated version of it [35], the results can be compared. In line with our results, several authors also found that patients with myofascial pain or painful TMD showed a higher degree of somatization [41,43,49]. Finally, the OPPERA study [19] concluded that the somatic symptoms increase the risk of suffering from TMD and are one of the most important psychological predictors.

In the present study, the coping styles to stress situations were thoroughly investigated by employing the CRIA inventory. The participants suffering from just TMD myalgia presented diminished coping strategies compared to the control group. Interestingly, for some strategies, a significant reduction appeared for the TMD myalgia group without migraine in comparison to both the migraine and the TMD groups. Particularly, the TMD patients without migraine presented significantly fewer levels of positive reappraisal, seeking for guidance, cognitive avoidance, search for alternative rewards, and emotional discharge, and a general lack of coping strategies regardless of whether they were approached or avoidance strategies. The literature on stress coping is quite heterogeneous. However, passive vs. active, avoidance vs. approach strategies, and adaptive vs. maladaptive coping styles have been often considered as corresponding concepts [50]. Previous studies generally found greater use of maladaptive/avoidance strategies (such as acceptance and resignation) and/or less use of adaptive behaviors for TMD patients [51,52,53], while healthy bruxers without TMD presented a larger use of adaptative coping strategies in comparison to controls [54]. Nonetheless, it was found that patients with muscular TMD pain showed a more active coping style, with a tendency for minor use of humor than articular TMD pain patients [55]. Altogether, it can be hypothesized that both the absence of coping strategies and/or the presence of non-adaptive strategies could be related to TMD. 

Regarding the coping style in migraine patients, they presented coping styles similar to those of the controls. This was different from what was expected, since a previous study reported that migraine patients may differ in how they cope compared to healthy adults, as they tend to use more internal styles to cope with stress instead of seeking social support [56]. Nonetheless, the presence of TMD was not controlled in this study. Therefore, further research is required on coping styles and migraine patients controlling for TMD, to clarify to which extent maladaptive coping is related to migraine.

### 4.1. Limitations and Strengths

It should be noticed that 20 out of 80 of the explored migraine patients did not present also TMD and just 2 of them experienced chronic migraine. Therefore, although further research is required, it can be argued that the presence of TMD myalgia might be an important factor for migraine chronification, in line with previous literature [9,46,57]. Furthermore, since the participants were consecutively recruited and clinically assessed, the sample size for each group was smaller for the TMD−/Migraine+ and TMD+/Migraine− patients than for the TMD+/Migraine+ ones, also reflecting the high comorbidity between TMD and migraine [14,27,58]. Additionally, the TMD−/Migraine+ group included only 10% of chronic migraine patients, while the TMD+/Migraine+ group included 56% of chronic migraine patients. Although, due to sample limitations, it was out of the scope of this article, as requested by a reviewer, the psychological variables of episodic and chronic migraine patients were statistically compared. Significant effects were observed exclusively for somatization (Δ = 3.6; *p* < 0.05), together with a strong trend for anxiety symptoms (Δ = 2.6; *p* = 0.06) in the BSI-18 questionnaire. Therefore, differences between the TMD−/Migraine+ and TMD+/Migraine+ groups were expected, since severe pain is more likely to appear in chronic migraine patients than in episodic migraine ones [59,60]. However, differences between these two groups were not observed. Nonetheless, to entirely rule out that pain and symptoms severity do not play a role in the present findings, further research including the evaluation of a severity index is required.

The relations between TMD and migraine are widely discussed in the literature [9,14,58,61]; however, little is known about the contribution of psychological factors to this association and the possible existence of different psychological profiles for each pathology. Therefore, the data obtained in this study might be a valuable contribution in this sense.

### 4.2. Highlights and Future Directions

Suffering a stressful situation together with the lack of coping strategies in the absence of migraine could be a risk factor for suffering TMD, while migraine seems to be more related to anxiety traits and depression. Although this study focused on TMD myalgia, due to sample limitations, it was not possible to distinguish between chronic and episodic migraine with and without TMD myalgia. Furthermore, although the present study was designed to obtain a thorough evaluation of psychological variables, it was not possible to disentangle to what extent the observed differences in the psychological profiles were consequences of or risk factors for migraine and/or TMD. Therefore, it is necessary to continue investigating this field using a thorough psychological assessment, also including personality questionnaires and implementing longitudinal designs in large and representative samples comparing similar numbers of patients per group, while controlling for severity, the presence of headaches, the migraine types, and other comorbidities among pathologies. Nonetheless, this study provides valuable data about what to expect in terms of psychological variables in TMD and migraine patients with and without comorbidities.

## 5. Conclusions

According to the present study data, situational anxiety (transient emotional state), together with a lack of coping strategies, could be more associated with TMD myalgia, while the anxiety trait, as a more stable and long-lasting emotional state, together with depression might be more related to migraine. Therefore, although similarities exist, different psychological profiles seem to be associated with TMD myalgia and migraine. These different profiles should be taken into consideration in patients’ treatment and management. Furthermore, future longitudinal studies would help to disentangle if TMD myalgia patients with greater levels of anxiety trait and depression might be more prone to develop migraine and to unravel to what extent severity affects the psychological profiles.

## Figures and Tables

**Table 1:** Sample characteristics depending on the group.

	TMD−/MIGRAINE−	TMD−/MIGRAINE+	TMD+/MIGRAINE−	TMD+/MIGRAINE+
Participants (n)	39	20	23	60
Females (males)	27 (12)	16 (4)	19 (4)	52 (8)
Mean Age	37.8	34.2	35.1	42
Episodic Mig.	NA	18	NA	26
Chronic Mig.	NA	2	NA	34
Local myalgia	NA	NA	2	13
Myofascial pain	NA	NA	14	6
Myofascial pain with referral	NA	NA	7	34
Headache attributed to TMD	NA	NA	0	5
Arthralgia	NA	NA	0	2

Sample descriptions: number of participants per group in each category except for “mean age”; NA = did not apply, Mig. = migraine, TMD = temporomandibular disorders.

## Data Availability

The data presented in this study are available on request from the corresponding authors.

## Supplementary Materials

The following supporting information can be downloaded at: https://www.mdpi.com/article/10.3390/ijerph20021545/s1, Table S1: Migraine (+/−) × TMD (+/−) 2 × 2 MANOVA results; Table S2: Post-hoc analyses for significant Migraine × TMD interactions.
