# Supplementary material for: Psychological and Behavioral Factors Involved in Temporomandibular Myalgia and Migraine: Common but Differentiated Profiles
Source: Int J Environ Res Public Health. 2023 Jan 14;20(2):1545. doi: 10.3390/ijerph20021545 (PMC9863553; doi:10.3390/ijerph20021545)
Supplement: Supplementary file 1 [file ijerph-20-01545-s001.zip › ijerph-2142945-supplementary.pdf]

Supplementary Material

Table S1. Migraine (+/-) x TMD (+/-) 2 x 2 MANOVA results.

			Mean	Standard desviation (SD)	degrees of freedom	F	p (sig)	Effect size (ηp2)	Power (θ)
Anxiety State (STAI)	Migraine	+	19.39	12.5	1.138	2.76	0.099	0.02	0.37
		-	14.61	9.1					
	TMD	+	19.65	11.53	1.138	4.98	**0.027**	0.03	0.6
		-	14	10.32					
	Migraine * TMD				1.138	1.48	0.226	0.01	0.23
Anxiety Trait (STAI)	Migraine	+	22.35	13.09	1.138	3.24	*0.064*	0.02	0.43
		-	17.42	9.35					
	TMD	+	21.86	12.02	1.138	1.29	0.258	0.009	0.2
		-	17.86	11.23					
	Migraine * TMD				1.138	0.33	0.566	0.002	0.09
Depression State (ST/DEP)	Migraine	+	19.1	7.18	1.138	4.52	**0.035**	0.032	0.56
		-	16.24	4.43					
	TMD	+	18.81	6.57	1.138	1.58	0.212	0.011	0.24
		-	16.51	5.62					
	Migraine * TMD				1.138	0.37	0.545	0.003	0.09
State Euthymia (ST/DEP)	Migraine	+	11.9	4.38	1.138	3.28	0.07	0.023	0.44
		-	10.23	3.29					
	TMD	+	11.83	4.17	1.138	2.4	0.12	0.02	0.34
		-	10.24	3.64					
	Migraine * TMD				1.138	0.52	0.47	0.004	0.11
State Dysthymia (ST/DEP)	Migraine	+	7.2	3.4	1.138	5.7	**0.018**	0.04	0.66
		-	5.94	1.38					
	TMD	+	6.92	3.01	1.138	0.14	0.71	0.001	0.07
		-	6.27	2.37					
	Migraine * TMD				1.138	0.01	0.92	0	0.05
Depression Trait (ST/DEP)	Migraine	+	17.76	6.54	1.138	3.52	0.063	0.03	0.47
		-	15.61	4.63					
	TMD	+	17.29	6.09	1.138	0.102	0.75	0.001	0.06
		-	16.17	5.5					
	Migraine * TMD				1.138	0.005	0.95	0	0.05
Trait Euthymia (ST/DEP)	Migraine	+	10.14	3.96	1.138	0.99	0.32	0.007	0.16
		-	9.37	3.5					
	TMD	+	10.02	3.93	1.138	0.17	0.69	0.001	0.07
		-	9.49	3.56					
	Migraine * TMD				1.138	0.19	0.67	0.001	0.07
Trait Dysthymia (ST/DEP)	Migraine	+	7.63	3.05	1.138	5.26	**0.023**	0.04	0.62
		-	6.4	1.8					
	TMD	+	7.39	2.77	1.138	0.33	0.57	0.002	0.091
		-	6.68	2.42					
	Migraine * TMD				1.138	0.1	0.75	0.001	0.06
BSI Somatization	Migraine	+	6.58	5.28	1.138	14.43	**0**	0.09	0.97
		-	2.79	3.01					
	TMD	+	6.16	5.12	1.138	4.78	**0.03**	0.035	0.58

		-	3.19	3.72					
	Migraine * TMD				1.138	0.17	0.68	0.001	0.07
BSI Depression	Migraine	+	5.14	5.99	1.138	8.59	**0.004**	0.06	0.83
		-	2.52	3.05					
	TMD	+	4.31	5.29	1.138	0.09	0.76	0.001	0.06
		-	3.54	4.79					
	Migraine * TMD				1.138	0.19	0.66	0.001	0.072
BSI Anxiety	Migraine	+	5.94	5.63	1.138	4.74	**0.031**	0.033	0.58
		-	3.58	3.69					
	TMD	+	5.59	5.48	1.138	0.89	0.35	0.006	0.16
		-	3.95	4.1					
	Migraine * TMD				1.138	0.44	0.5	0.003	0.1
BSI global severity index	Migraine	+	17.65	15.38	1.138	10.49	**0.002**	0.07	0.89
		-	8.89	8.69					
	TMD	+	16.06	14.45	1.138	0.96	0.33	0.007	0.16
		-	10.68	11.64					
	Migraine * TMD		13.82	13.58	1.138	0.31	0.58	0.002	0.09
CRI-Logical Analysis (LA)	Migraine	+	10.63	3.98	1.138	0.26	0.61	0.002	0.08
		-	10.21	4.36					
	TMD	+	10.34	4.18	1.138	0.29	0.59	0.002	0.084
		-	10.54	4.12					
	Migraine * TMD				1.138	3.97	0.006	0.028	0.51
CRI-Positive Reappraisal (PR)	Migraine	+	11.55	4.07	1.138	1.9	0.17	0.014	0.28
		-	10.45	4.2					
	TMD	+	11.07	4.05	1.138	0.52	0.47	0.004	0.11
		-	11.09	4.32					
	Migraine * TMD				1.138	9.95	**0.002**	0.067	0.88
CRI-Seeking Guidance (SG)	Migraine	+	9.99	3.6	1.138	0.41	0.521	0.003	0.1
		-	9.34	4.1					
	TMD	+	9.76	3.82	1.138	0.068	0.79	0	0.06
		-	9.63	3.86					
	Migraine * TMD				1.138	11.3	**0.001**	0.08	0.92
CRI-Problem Solving (PS)	Migraine	+	11.99	4.01	1.138	0.01	0.91	0	0.05
		-	11.84	3.83					
	TMD	+	11.86	3.84	1.138	0.154	0.69	0.001	0.07
		-	12.02	4.06					
	Migraine * TMD				1.138	4.16	**0.043**	0.03	0.53
CRI- Cognitive Avoidance (CA)	Migraine	+	8.54	3.62	1.138	6.95	**0.009**	0.05	0.75
		-	6.66	4.47					
	TMD	+	7.86	3.96	1.138	0.41	0.53	0.003	0.1
		-	7.53	4.33					
	Migraine * TMD				1.138	1.34	0.25	0.01	0.21
CRI- Acceptance/Resignation (AR)	Migraine	+	7.28	3.61	1.138	2.43	0.12	0.02	0.34
		-	6.05	3.56					
	TMD	+	7.02	3.68	1.138	0.13	0.72	0.001	0.064
		-	6.34	3.55					

	Migraine * TMD				1.138	1.71	0.19	0.01	0.25
CRI - Seeking Alternative Reward (SA)	Migraine	+	7.78	3.85	1.138	1.15	0.29	0.008	0.19
		-	7.24	3.96					
	TMD	+	7.24	3.98	1.138	2.53	0.114	0.02	0.352
		-	7.97	3.76					
	Migraine * TMD				1.138	5.74	**0.02**	0.04	0.66
CRI - Emotional Discharge (ED)	Migraine	+	6.6	3.98	1.138	2.62	0.11	0.02	0.36
		-	5.39	3.42					
	TMD	+	6.22	3.78	1.138	0.05	0.82	0	0.06
		-	5.86	3.8					
	Migraine * TMD				1.138	3.93	0.05	0.03	0.5

Table S2. Post-Hoc analyses for significant Migraine x TMD interactions.

**Tukey Post-Hoc Testing**					
Dependent variable			**Mean difference**	**Dev. Error**	**Sig. (p)**
CRI- Logical Analysis	Control Group (TMD-/Migraine-)	TMD-/Migraine+	1.1231	1.1324	0.754
		TMD+/Migraine-	1.9231	1.0825	0.289
		TMD+/Migraine+	0.0231	0.8469	1
	TMD-/Migraine+	Control group	-1.1231	1.1324	0.754
		TMD+/Migraine-	0.8	1.2588	0.92
		TMD+/Migraine+	-1.1	1.0631	0.729
	TMD+/Migraine-	Control group	-1.9231	1.0825	0.289
		TMD-/Migraine+	-0.8	1.2588	0.92
		TMD+/Migraine+	-1.9	1.0097	0.241
CRI-Positive Reappraisal	Control Group (TMD-/Migraine-)	TMD-/Migraine+	1.3128	1.1026	0.634
		TMD+/Migraine-	2.8606	1.054	**0.037**
		TMD+/Migraine+	-0.4872	0.8246	0.935
	TMD-/Migraine+	Control group	-1.3128	1.1026	0.634
		TMD+/Migraine-	1.5478	1.2258	0.588
		TMD+/Migraine+	-1.8	1.0352	0.308
	TMD+/Migraine-	Control group	-2.8606	1.054	**0.037**
		TMD-/Migraine+	-1.5478	1.2258	0.588
		TMD+/Migraine+	-3.3478	0.9832	**0.005**
CRI- Seeking Guidance and Support	Control Group (TMD-/Migraine-)	TMD-/Migraine+	1.8564	1.0192	0.268
		TMD+/Migraine-	2.4738	0.9743	*0.058*
		TMD+/Migraine+	-0.2603	0.7622	0.986
	TMD-/Migraine+	Control group	-1.8564	1.0192	0.268
		TMD+/Migraine-	0.6174	1.133	0.948
		TMD+/Migraine+	-2.1167	0.9568	0.125
	TMD+/Migraine-	Control group	-2.4738	0.9743	*0.058*
		TMD-/Migraine+	-0.6174	1.133	0.948
		TMD+/Migraine+	-2.7341	0.9088	**0.016**
CRI- Problem Solving	Control Group (TMD-/Migraine-)	TMD-/Migraine+	1.3872	1.0729	0.569
		TMD+/Migraine-	1.748	1.0256	0.325
		TMD+/Migraine+	0.2038	0.8024	0.994
	TMD-/Migraine+	Control group	-1.3872	1.0729	0.569
		TMD+/Migraine-	0.3609	1.1927	0.99
		TMD+/Migraine+	-1.1833	1.0073	0.644
	TMD+/Migraine-	Control group	-1.748	1.0256	0.325
		TMD-/Migraine+	-0.3609	1.1927	0.99
		TMD+/Migraine+	-1.5442	0.9567	0.374
CRI- Cognitive Avoidance	Control Group (TMD-/Migraine-)	TMD-/Migraine+	-1.0962	1.1051	0.754
		TMD+/Migraine-	1.3278	1.0564	0.592
		TMD+/Migraine+	-1.4795	0.8265	0.282
	TMD-/Migraine+	Control group	1.0962	1.1051	0.754
		TMD+/Migraine-	2.4239	1.2285	0.203
		TMD+/Migraine+	-0.3833	1.0375	0.983
	TMD+/Migraine-	Control group	-1.3278	1.0564	0.592
		TMD-/Migraine+	-2.4239	1.2285	0.203
		TMD+/Migraine+	-2.8072	0.9854	**0.026**

CRI- Acceptance or Resignation	Control Group (TMD-/Migraine-)	TMD-/Migraine+	-0.1679	0.9886	0.998
		TMD+/Migraine-	0.6299	0.945	0.909
		TMD+/Migraine+	-1.2679	0.7393	0.32
	TMD-/Migraine+	Control group	0.1679	0.9886	0.998
		TMD+/Migraine-	0.7978	1.099	0.887
		TMD+/Migraine+	-1.1	0.9281	0.637
	TMD+/Migraine-	Control group	-0.6299	0.945	0.909
		TMD-/Migraine+	-0.7978	1.099	0.887
		TMD+/Migraine+	-1.8978	0.8815	0.142
CRI - Seeking Alternative Reward	Control Group (TMD-/Migraine-)	TMD-/Migraine+	0.9321	1.0498	0.811
		TMD+/Migraine-	2.8038	1.0036	**0.03**
		TMD+/Migraine+	0.3654	0.7851	0.966
	TMD-/Migraine+	Control group	-0.9321	1.0498	0.811
		TMD+/Migraine-	1.8717	1.1671	0.38
		TMD+/Migraine+	-0.5667	0.9856	0.939
	TMD+/Migraine-	Control group	-2.8038	1.0036	**0.03**
		TMD-/Migraine+	-1.8717	1.1671	0.38
		TMD+/Migraine+	-2.4384	0.9361	**0.049**
CRI - Emotional Discharge	Control Group (TMD-/Migraine-)	TMD-/Migraine+	0.2487	1.0224	0.995
		TMD+/Migraine-	1.5139	0.9774	0.411
		TMD+/Migraine+	-0.9513	0.7646	0.6
	TMD-/Migraine+	Control group	-0.2487	1.0224	0.995
		TMD+/Migraine-	1.2652	1.1366	0.682
		TMD+/Migraine+	-1.2	0.9599	0.596
	TMD+/Migraine-	Control group	-1.5139	0.9774	0.411
		TMD-/Migraine+	-1.2652	1.1366	0.682
		TMD+/Migraine+	-2.4652	0.9117	**0.038**
